# Endometriosis Lowers the Cumulative Live Birth Rates in IVF by Decreasing the Number of Embryos but Not Their Quality

**DOI:** 10.3390/jcm9082478

**Published:** 2020-08-01

**Authors:** Lisa Boucret, Pierre-Emmanuel Bouet, Jérémie Riou, Guillaume Legendre, Léa Delbos, Hady El Hachem, Philippe Descamps, Pascal Reynier, Pascale May-Panloup

**Affiliations:** 1 Reproductive Biology Unit, Angers University Hospital, 49000 Angers, France; pamaypanloup@chu-angers.fr; 2 Department of Reproductive Medicine, Angers University Hospital, 49000 Angers, France; pierreemmanuel.bouet@chu-angers.fr (P.-E.B.); guillaume.legendre@chu-angers.fr (G.L.); Lea.Delbos@chu-angers.fr (L.D.); phdescamps@chu-angers.fr (P.D.); 3 MINT, Angers University, INSERM 1066, CNRS 6021, IBS–CHU, 49000 Angers, France; jeremie.riou@univ-angers.fr; 4 Department of Reproductive Medicine, Saint Joseph Fertility Center, 1100 Beirut, Lebanon; hadyhachem@hotmail.com; 5 Department of Biochemistry and Genetics, Angers University Hospital, 49000 Angers, France; PaReynier@chu-angers.fr

**Keywords:** endometriosis, embryo quality, embryo morphology, IVF outcomes

## Abstract

Endometriosis and infertility are closely linked, but the underlying mechanisms are still poorly understood. This study aimed to evaluate the impact of endometriosis on in vitro fertilization (IVF) parameters, especially on embryo quality and IVF outcomes. A total of 1124 cycles with intracytoplasmic sperm injection were retrospectively evaluated, including 155 cycles with endometriosis and 969 cycles without endometriosis. Women with endometriosis had significantly lower ovarian reserve markers (AMH and AFC), regardless of previous ovarian surgery. Despite receiving significantly higher doses of exogenous gonadotropins, they had significantly fewer oocytes, mature oocytes, embryos, and top-quality embryos than women in the control group. Multivariate analysis did not reveal any association between endometriosis and the proportion of top-quality embryo (OR = 0.87; 95% CI [0.66–1.12]; *p* = 0.3). The implantation rate and the live birth rate per cycle were comparable between the two groups (*p* = 0.05), but the cumulative live births rate was significantly lower in in the endometriosis group (32.1% versus 50.7%, *p* = 0.001), as a consequence of the lower number of frozen embryos. In conclusion, endometriosis lowers the cumulative live birth rates by decreasing the number of embryos available to transfer, but not their quality.

## 1. Introduction

Endometriosis is a complex gynecological disease whose pathogenesis is still widely debated. It can affect several anatomical sites such as the ovaries, the peritoneum, the bladder and the rectum. This disease is associated with infertility, dysmenorrhea, and chronic pelvic pain and can impair quality of life in more than one way [1,2,3]. The prevalence of endometriosis is difficult to evaluate in the general population, but best estimates conclude that approximately 10% of women of reproductive age are afflicted with endometriosis [4]. Several pathophysiological mechanisms have been proposed to explain the infertility caused by endometriosis, such as pelvic adhesions caused by the lesions, especially those involving the fallopian tubes; pelvic inflammation that could disrupt fertilization and implantation; and an impairment of ovarian function [4,5]. The latter is thought to be linked to a decrease in the ovarian reserve, whether following repeated ovarian surgeries, or as a direct consequence of endometriomas. Indeed, according to Kitajima’s theory of follicular burnout [6], endometriomas can cause focal inflammation, structural damage to the adjacent cortex, massive fibrosis, and stromal loss specifically affecting primordial follicles. Focal loss of follicular density may cause dysregulation of folliculogenesis, with increased recruitment and atresia acting in a vicious circle, leading to a loss of the follicular capital, resulting in an overall decline in ovarian reserve. The negative effect of endometriosis on ovarian reserve and response to controlled ovarian stimulation (COS) for in vitro fertilization (IVF) has been shown by different studies [7,8,9,10,11,12], but the impact of endometriosis on the oocyte and embryo quality is still debated [13,14,15,16]. In this context, the aim of this study was to assess the impact of endometriosis on IVF cycles parameters, especially on embryo quality and IVF outcomes.

## 2. Materials and Methods

### 2.1. Study Design

We performed a retrospective cohort study based on all patients undergoing intracytoplasmic sperm injection (ICSI) treatment at the reproductive medicine center of Angers hospital (France), between January 2014 and March 2018. Couples were referred for ICSI because of male factor infertility, prior fertilization failure, or endometriosis. Indeed, at our center, we perform ICSI for patients with confirmed endometriosis, in order to optimize fertilization [16,17]. The diagnosis of endometriosis was confirmed by ultrasound, Magnetic Resonance Imaging (MRI), or following abdomino-pelvic surgery. Endometriosis was scored according to the revised classification of the American Society of Reproductive Medicine (ASRM) [18]. There was a history of abdominopelvic surgery for all patients with stage I-II endometriosis. For stage III or greater endometriosis, the rASRM classification was if necessary extrapolated from the imaging techniques.

In our center, the indications for endometriosis surgery before or after Assisted Reproductive Technology (ART) in case of endometrioma (OMA) or deep infiltrating endometriosis (DIE) respect the French recommendations published in 2018 and inspired by the recent literature on the subject [19]. The benefit of surgical treatment of endometriomas smaller than 6 cm compared to abstention was not proven on the results of pregnancy after IVF [19]. In case of pain and/or endometrioma greater than or equal to 6 cm, laparoscopic cystectomy is the reference technique for the treatment of endometriomas. In the case of recurrent cysts less than 8 cm, an ultrasound-guided aspiration with ethanol sclerotherapy may be indicated. DIE surgery provides pregnancy rates broadly comparable to the pregnancy rates obtained by ART in non-operated patients. To date, there is no data from comparative studies with sufficient level of evidence to formally recommend surgery or ART first line in women with DIE. If the main symptom is infertility and not pain, we perform first line ART rather than a surgical treatment due to the potential preoperative difficulties and to the risks of postoperative complications. After ART, surgical management of DIE can be performed in case of chronic pain associated with an impaired quality of life and after failure of medical treatment.

The study group included all patients with endometriosis, the control group included all other non-endometriosis patients. All patients had a baseline fertility assessment prior to IVF that included basal day 3 FSH and anti-Müllerian hormone (AMH) level, as well as a pelvic ultrasound for Antral Follicle Count (AFC). Cycles were performed routinely without any additional intervention required by this study. All participants did not object to the processing of their personal data.

### 2.2. Controlled Ovarian Stimulation

Patients were stimulated either with a long gonadotropin-releasing hormone (GnRH) agonist or an antagonist protocol. At our center, we offer the long agonist protocol first line for patients with endometriosis, based on studies showing better pregnancy rates [20]. For the long agonist protocol, pituitary desensitization was achieved with daily triptorelin 0.1 mg, started on the twenty first day of the previous cycle. In the antagonist protocol, GnRH antagonist was added on the sixth day of stimulation. Patients received either recombinant FSH or FSH and LH, and the initial dose of gonadotropins was based on the patient’s age, ovarian reserve, body mass index (BMI), and, if applicable, previous responses to stimulation. All patients had serial ultrasounds and blood measurements to monitor the follicular growth, and ovulation was triggered with 250 μg recombinant hCG (Ovitrelle^®^, Merck, Lyon, France) when at least 4 follicles reached a size greater than 17 mm. Oocyte retrieval was scheduled 36 h after the hCG injection and performed under general anesthesia.

### 2.3. Fertilization and Embryo Culture

The cumulus-oocyte complexes were washed in multiple dishes with Flushing medium^®^ (Origio, Malov, Denmark) and denuded one hour after, using hyaluronidase (FertiPro, Beernem, Belgium) and mechanical stripping. Sperm preparation was achieved by a 40/80% discontinuous density gradient using PureSperm^®^ (Nidacon, Mölndal, Sweden), or by washing with FertiCult^®^ IVF medium (FertiPro, Beernem, Belgium) in cases of cryptozoospermia. Metaphase II (MII) oocytes, i.e., oocytes that expelled the first polar body 1 h after oocyte collection, were injected with spermatozoa for the ICSI procedure. Fertilization was assessed 18 h post-injection. Embryos were cultured in Global medium^®^ (LifeGlobal, Guilford, USA) with 10% of Human Serum Albumin (Cooper-Surgical, Trumbull, CT, USA) at 37 °C under an atmosphere containing 6% CO2 and 7% oxygen and were conventionally observed, once every day, until the transfer or the cryopreservation. We used the ESHRE classification system to assess embryo morphology at the cleavage stage and Gardner’s classification at the blastocyst stage [21]. Only the embryo morphology at day 2 was retained for this study, since the majority of our transfers were at this cleavage stage.

### 2.4. Embryo Transfer and Freezing

Embryo transfer was generally performed on day 2 or day 3 after the oocyte retrieval for a first or second cycle and otherwise on day 5. Only one or two embryos were transferred, depending on the patient age, the cycle rank, and the embryo quality, but irrespective of the endometriosis status of the patient. Overall, for the first transfer in women under 35 years old, we transferred only one embryo if embryo freezing was possible. For women over 35, or for younger women undergoing attempts ranked above one, we transferred two embryos. Vaginal micronized progesterone (600 mg/day) was administered for luteal phase support from the day of oocyte retrieval and continued till twelve weeks gestational age (GA). Biological pregnancy was initially diagnosed by a serum bhCG level above 100 IU/L, which was tested 14 days after oocyte retrieval. A vaginal ultrasound was performed at 7 weeks GA to confirm a clinical pregnancy (defined as a positive fetal heartbeat).

All remaining good quality embryos were cryopreserved. Cleavage stage embryos were cryopreserved by slow freezing (Origio, Malov, Denmark) prior to September 2016 and by vitrification (Fujifilm-Irvine Scientific, Santa Ana, California, USA) afterwards, while blastocysts were always cryopreserved by vitrification (Irvine), according to the manufacturer’s recommendations. Five hundred eighty-one frozen embryos transfers were performed, mostly substituted cycles (>90% cycles). Natural or stimulated cycles could be used in cases of recurrent implantation failure.

### 2.5. Outcomes

Our primary outcome measures were the number of oocytes, the maturity rate (proportion of MII oocytes over to the total number of oocytes retrieved), the number of embryos, the cleavage rate (number of day-3 embryos divided by number of MII oocytes), the proportion of top-quality embryos (proportion of embryos with the highest score on day 2—grade 1 according to the ESHRE consensus—over to the number of embryos obtained), the usable embryo yield (proportion of embryos either transferred or cryopreserved), and the number of frozen embryos. The secondary outcome measures were the implantation rate (number of gestational sacs with a fetal heartbeat divided by the number of embryos transferred), the live births rate (delivery after 22 weeks GA) and the cumulative live births rate (using fresh and frozen embryos of different cycles for a same patient).

### 2.6. Statistical Analysis

Categorical variables were compared using χ^2^ test, while continuous variables were compared using the non-parametric Mann–Whitney U-test. All the calculations were computed using R [22]. Because of its non-normal distribution, multivariate analysis of embryo quality was performed using Poisson regression [23]. Differences were considered significant at *p* < 0.05 (*).

### 2.7. Ethical Approval

The study was conducted according to the ethical standards of the Helsinki Declaration and its later amendments and with the approval of the Ethics Committee of the Angers University Hospital, France (Number 2020/66). All the data were anonymously collected from the local database, in accordance with the French National Commission for Information and Liberties (ar20-0029v0).

## 3. Results

### 3.1. Patient Characteristics

Among the 1124 cycles reported, 155 (13.8%) ICSI cycles concerned 84 patients with endometriosis and 969 cycles (86.2%) referred to 590 patients in the control group. Baseline characteristics are described in Table 1. There were no significant differences in the duration of infertility and in the cycle rank between the two groups. The mean number of cycles was 1.8 for patients with endometriosis and 1.6 for patients without endometriosis. Body mass index (BMI) was significantly lower in the endometriosis group (23.4 ± 4.3) compared to the control group (24.6 ± 5.3) (*p* = 0.03). As expected, the total motile sperm count was significantly higher in the endometriosis group (56.3 ± 80.4 millions vs. 18.6 ± 41.8 millions, *p* < 0.0001) because of the inclusion of male factor infertility in the control group. Mean age and tobacco use, known as confounder of ovarian reserve markers, were comparable between the two groups. Patients in the endometriosis group had a significantly lower ovarian reserve, with a significantly lower mean serum AMH level (2.7 ± 2.3 ng/mL vs 3.9 ± 3.9 ng/mL, *p* = 0.0002) and a significantly lower mean AFC (16 ± 10 vs 20 ± 11 *p* < 0.0001). However, day 3 serum FSH levels were comparable between the two groups (*p* = 0.1).

### 3.2. Stimulation Protocols

Patients in the endometriosis group required significantly more FSH (2819 ± 1109 IU) during stimulation than in the control group (2342 ± 986 IU) (*p* < 0.0001). As expected, the long agonist protocol was more often prescribed in the endometriosis group than in the control group (39.4% vs. 9.9%, *p* < 0.0001). The type of gonadotropins used (FSH vs FSH + LH) did not differ between the 2 groups (*p* = 0.5) (Table 1).

### 3.3. IVF Outcomes

The IVF parameters are summarized in Table 2. Women in the endometriosis group had significantly fewer oocytes retrieved (7.0 ± 4.3 vs. 9.7 ± 6.4, *p* < 0.0001) and MII oocytes (4.8 ± 3.5 vs. 6.9 ± 5.0, *p* < 0.0001) compared to the control group, but the oocyte maturity rate was comparable between the two groups (68.6 ± 24.5 vs. 71.1 ± 23.7, *p* = 0.2). The total number of embryos (3.5 ± 2.9 vs. 4.8 ± 3.9, *p* = 0.0006) and top-quality embryos (0.5 ± 0.8 vs. 0.7 ± 1.1, *p* = 0.01) was significantly lower in the endometriosis group compared to the control group, but there were no differences in the cleavage rate. The mean number of embryos with late cleavage (cleavage beginning on day 3) and the mean number of transferred embryos did not differ between the two groups. We found a trend towards a lower proportion of top-quality embryos in the endometriosis group, but the difference did not reach clinical significance (*p* = 0.06), while the proportion of usable embryos was comparable between the two groups (*p* = 0.1). A multivariate Poisson regression was performed to adjust for baseline differences in BMI, AMH, AFC, protocol, total dose of FSH required, and number of oocytes retrieved. The analysis did not reveal any significant association between endometriosis and the proportion of top-quality embryos (OR = 0.87; 95% CI (0.66–1.12); *p* = 0.3).

### 3.4. IVF Success Rates

Despite a marginal trend towards significance (*p* = 0.05), the implantation rate (17.3% vs. 23.6%) and the live birth rate per cycle (16.1% vs. 23.2%) were comparable between the endometriosis and the control groups. Patients in the endometriosis group had a significantly lower number of frozen embryos (0.8 ± 1.6 vs 1.5 ± 2.2, *p* = 0.0003), frozen embryo transfers (0.32 vs. 0.55, *p* = 0.001) and thus had a significantly lower cumulative live birth rate (32.1% vs. 50.7%, *p* = 0.001) compared to the control group (Table 2).

### 3.5. Subgroup Analysis

In order to further investigate the potential impact of the endometriosis stage on IVF outcomes, we performed a subgroup analysis in the endometriosis group and compared patients with stage I–II (*n* = 28, 18.1% of cycles) to patients with stage III or greater endometriosis (*n* = 127, 81.9% of cycles) (Table 3). The patients’ characteristics were comparable between the two groups, and as expected, there was a significantly higher prevalence of ovarian surgery in the stage III or greater group compared to stage I–II (49.6% vs. 7.1%, *p* < 0.0001). All the IVF cycle characteristics and outcomes were comparable between the two groups. Pelvic adhesions were described in 23% of all patients, but the description of types and degree of adhesions was not always available.

Finally, in order to assess the impact of endometriosis on the ovarian reserve and the IVF outcomes irrespective of surgery, we identified a subgroup of patients with confirmed endometriosis but without previous ovarian surgery (*n* = 90). When compared to the control group, these patients had significantly lower ovarian reserve parameters (AMH, AFC, and FSH), required significantly higher doses of gonadotropins, and had a significantly lower number of oocytes retrieved and embryos. However, the implantation and live birth rates were comparable with the control group (Table 4).

## 4. Discussion

Our study aimed to assess the impact of endometriosis on IVF outcomes, especially on embryo quality and live birth rates. We found that women with endometriosis had a significantly lower number of oocytes and mature oocytes retrieved despite receiving higher gonadotropins doses, and a significantly lower number of embryos and top-quality embryos. The proportion of top-quality embryos and the proportion of usable embryos did not differ significantly between the two groups. The live birth rates per cycle were comparable between the two groups, but patients with endometriosis had a significantly lower cumulative live birth rate, because of a significantly lower number of frozen embryo transfers.

Pelvic organ adhesion and distortion, as well as defective endometrial receptivity, have long been recognized as causes of infertility in patients with endometriosis [5]. However, the impact of endometriosis on oocyte quality and embryo development is still subject to debate [24], with most studies and meta analyses focusing on its impact on the clinical outcomes instead [25,26,27,28,29,30]. One way to investigate the impact of endometriosis on oocyte competence is through the analysis of the follicular fluid. Indeed, follicular fluid is an active microenvironment which has a critical role in oocyte growth and maturation. Differences in the concentration of inflammatory cytokines [31,32,33] and oxidative stress markers [34,35] in follicular fluid have been reported between women with and without endometriosis. Endometriosis could also affect the metabolic processes, thereby altering the microenvironment of the follicle and the oocyte development. Indeed, metabolomic signatures related to endometriosis have been identified in the follicular fluid [36,37,38], and in other biological fluids [39,40], despite some reports suggesting the opposite [41]. One study reported that the follicular fluid of infertile women with endometriosis could impair nuclear maturation and promote meiotic anomalies in bovine oocytes matured in vitro [42]. At the transcriptomic level, Ferrero [43] recently identified differentially expressed genes in oocytes from women with ovarian endometriosis. Endometriosis could also be associated with mitochondrial dysfunction [44,45]. Xu [46] reported that oocytes from women with mild endometriosis exhibited decreased mitochondrial DNA copy number and abnormal mitochondrial structure. Altogether, these results suggest that endometriosis could impair oocyte microenvironment with deleterious consequences on oocyte and embryo quality. Studies assessing oocyte quality during ICSI procedures report frequent and significant morphological abnormalities in endometriosis patients [13,15,16,47]. Furthermore, in oocyte donation programs, recipients without endometriosis using oocytes from donors with endometriosis had significantly lower implantation rates when compared with recipients from donors without endometriosis [48,49]. Conversely, the implantation rate was not affected in recipients with endometriosis using oocytes from disease-free donors [49,50,51], even though these results are conflicting with a more recent study [52]. Altogether, these observations suggest that infertility in endometriosis patients may be related to disturbed oocyte microenvironment rather than an inappropriate endometrial environment.

In our study, patients in the endometriosis group had significantly a lower ovarian reserve (AMH and AFC) compared to the control group. Moreover, we identified a subgroup of women with confirmed endometriosis but without any previous surgery who also had a significantly lower ovarian reserve compared to the control group. This is in accordance with most studies that show a decreased ovarian reserve in patients with endometriosis, regardless of whether they had any previous ovarian surgery [53,54]. Endometrioma surgery can alter the ovarian reserve via the thermal, mechanical and devascularization injury [55], but endometriosis is also hypothesized to cause chronic inflammation and an altered immunomodulation in the ovary that could inherently damage the follicular reserve [53]. However, when we compared the effect of the endometriosis stage on the ovarian reserve, we did not find any significant difference, probably due to the small sample size of the stage I–II group. We also found a significantly lower number of oocytes retrieved and mature oocytes in women with endometriosis, despite the higher gonadotropin doses used. This is in accordance with the available literature and is explained by the lower ovarian reserve in these patients [7,8,9,10,11,12,56]. Another added factor that could contribute to the lower oocyte yield is the difficult access to the follicles during retrievals in the presence of large endometriomas [57]. The significantly lower number of embryos and top-quality embryos available is the direct consequence of the lower oocyte yield. It is worth noting that the significantly lower BMI in the endometriosis group did not affect the IVF parameters, since both groups had a BMI in the normal range (<25), and the parameters are only affected in obese women with BMI > 30 [58].

Despite having a significant decrease in the quantitative IVF parameters assessed, we did not find any detrimental impact of endometriosis on oocyte maturity rate and cleavage rate, and multivariate analysis did not show any association between endometriosis and embryo quality. Our findings on the embryo quality are in accordance with several reports [11,16,30,59,60,61,62,63,64,65] but contradictory to many others [10,13,66,67,68,69]. The available data regarding time-lapse morphokinetic parameters are also heterogeneous. Some studies suggest that embryos from endometriosis patients display altered early cell cycle synchronization [66,70,71] and duration [66], while others do not show any differences in morphokinetic variables between oocytes retrieved from an ovary with an endometrioma and sibling oocytes retrieved from the contralateral ovary [61].

We found a trend for decreased implantation and live birth rates in women with endometriosis, but the difference was not statistically significant. However, due to the lower number of frozen embryos in patients, the cumulative live birth rates were significantly lower in the endometriosis group. The impact of endometriosis on pregnancy and live birth rates following IVF remains controversial, with some studies confirming a significant negative impact and others reporting no effect [7,8,9,10,11,72,73]. The last published meta-analysis [29] showed significantly lower clinical pregnancy rates, but without any difference in live birth rates. A well-designed retrospective study based on the register of the Society for Assisted Reproductive Technology (SART) that included more than thirty nine thousand IVF cycles in women with endometriosis concluded that women with isolated endometriosis had similar or higher live birth rates compared to women with other diagnoses, whereas endometriosis women with concomitant diagnoses had lower live birth rates compared with other causes [12]. In our study, the majority of endometriosis patients had a concomitant diagnosis, as 49% of cases had a decreased ovarian reserve (AMH ≤ 2 ng/mL), 25% had concomitant male factor infertility (total motile sperm count < 5 millions).

Finally, our study failed to show any significant difference in embryo quality between the two groups. However, some values were close to the significance threshold and should therefore be interpreted with caution, especially since they may be subject to limits and biases. Indeed, we assessed embryo quality at the cleavage stage and were not able to assess the percentage of blastulation and blastocyst quality. Moreover, higher semen parameters (total motile sperm count) in the endometriosis group could have counteracted the potential effect of endometriosis on embryo quality, as a negative relationship between semen quality and embryo development is described in ICSI [74,75]. On the other hand, our study might suffer from a selection bias, since our center is a referral center for endometriosis management. Therefore, the women included in this study may have particularly severe forms of endometriosis (82% of stage III or greater), which could have impacted the IVF cycle outcomes. Despite the relatively large sample size, the retrospective and monocentric design of our study lowers the power of the conclusions and potentially underestimates the significance of certain factors. Finally, the operative notes, and the histological confirmation were not available for all the patients. It would be interesting to consider in further studies the effect of the different types of lesions (endometriomas, deep infiltrating endometriosis, adhesions) on the embryo quality and the IVF outcomes.

In conclusion, our study showed a significantly lower number of oocytes and embryos in women with endometriosis compared to patients with other causes of infertility but found no significantly difference in the embryo quality. The live birth rates per cycle were comparable between the two groups, but the cumulative live birth rate was significantly lower in women with endometriosis as a consequence of the lower number of frozen embryos. Endometriosis remains a complex disease that impairs quality of life [1,2,3] and women’s fertility and can impact the outcomes of assisted reproductive technologies in more than one way. Further studies are needed to improve our understanding of these mechanisms in light of the disease’s potential heterogeneity, in order to improve the management and the treatment outcomes.

## Figures and Tables

**Table 1:** Baseline characteristics and stimulation parameters in the endometriosis and control groups. Values are shown as n (%), percentage (%) or mean ± SD.

Variable	Endometriosis	Control	*p* Value
	Group (*n* = 155)	Group (*n* = 969)	
Duration of infertility (months)	51.8 ± 29.3	56.8 ± 32.4	0.09
Cycle rank	2.0 ± 1.0	1.9 ± 1.1	0.2
Age (years)	32.1 ± 3.5	32.6 ± 4.5	0.2
Body mass index (kg/m^2^)	23.4 ± 4.3	24.6 ± 5.3	0.03 *
Tobacco use (%)	34.3	35.9	0.9
AMH (ng/mL)	2.7 ± 2.3	3.9 ± 3.9	0.0002 *
Antral Follicular Count	16 ± 10	20 ± 11	<0.0001 *
Basal FSH (IU/L)	7.5 ± 2.8	7.0 ± 2.5	0.1
Total FSH administered (IU)	2819 ± 1109	2342 ± 986	<0.0001 *
Stimulation Protocol			
Antagonist (%)	58.7	88.5	<0.0001 *
Agonist (%)	39.4	9.9	
Natural (%)	1.9	1.5	
Gonadotropin type			
FSH (%)	77.3	79.4	0.5
FSH+LH (%)	22.7	20.6	
Total motile sperm count	56.3 ± 80.4	18.6 ± 41.8	<0.0001 *

* significant.

**Table 2:** IVF parameters and pregnancy outcomes of patients with endometriosis and patients in the control group. Values are shown as mean ± SD or percentage (%).

	Endometriosis	Control	*p* Value
	Group (*n* = 155)	Group (*n* = 969)	
Retrieved oocytes (*n*)	7.0 ± 4.3	9.7 ± 6.4	<0.0001 *
Metaphase II oocytes (*n*)	4.8 ± 3.5	6.9 ± 5.0	<0.0001 *
Oocyte maturity rate (%)	68.6 ± 24.5	71.1 ± 23.7	0.2
Embryos (*n*)	3.5 ± 2.9	4.8 ± 3.9	0.0006 *
Cleavage rate (%)	71.3 ± 29.2	68.4 ± 26.6	0.1
Transferred embryos (*n*)	1.2 ± 0.8	1.3 ± 0.7	0.6
Top-quality embryos (*n*)	0.5 ± 0.8	0.7 ± 1.1	0.01 *
Top-quality embryos (%)	12.8 ± 21.7	16.3 ± 25.2	0.06
Frozen embryos (*n*)	0.8 ± 1.6	1.5 ± 2.2	0.0003 *
Usable embryos (%)	61.6 ± 33.6	66.3 ± 28.1	0.1
Embryos with delayed cleavage (*n*)	0.15 ± 0.39	0.12 ± 0.39	0.2
Implantation rate (%)	17.3	23.6	0.05
Live birth rate/cycle (%)	16.1	23.2	0.05
Frozen embryo transfers (*n*)	0.32	0.55	0.001 *
Cumulative live birth rate/patient (%)	32.1	50.7	0.001 *

* significant.

**Table 3:** IVF cycle characteristics and outcomes, according to the stage of endometriosis. Values are shown as mean ± SD or percentage (%).

	Stage I–II	Stage III or Greater	
	(*n* = 28)	(*n* = 127)	*p* Value
Previous ovarian surgery (%)	7.1	49.6	< 0.0001 *
AMH (ng/mL)	2.7 ± 1.3	2.8 ± 2.5	0.4
Antral Follicular Count	17.6 ± 9.3	15.6 ± 10.2	0.3
Basal FSH (IU/L)	8.0 ± 2.6	7.3 ± 2.8	0.08
Total FSH administered (IU)	2718 ± 1121	2842 ± 1110	0.5
Retrieved oocytes (*n*)	7.3 ± 4.1	6.9 ± 4.4	0.7
Top-quality embryos (%)	18.6 ± 31.2	11.6 ± 18.9	0.6
Usable embryos (%)	71.6 ± 34.2	59.4 ± 33.2	0.1
Implantation rate (%)	11.1	18.7	0.4
Live birth rate/cycle (%)	11.1	17.2	0.4

* significant.

**Table 4:** IVF cycle characteristics and outcomes in endometriosis patients without endometrioma surgery, compared to control group. Values are shown as mean ± SD or percentage (%).

	Non-Endometriosis	Non-Operated Endometrioma	*p* Value
	(*n* = 969)	(*n*= 90)	
AMH (ng/mL)	3.9 ± 3.9	2.6 ± 2.7	0.0001 *
Antral Follicular Count	19.6 ± 10.6	16.7 ± 11.6	0.0004 *
Basal FSH (IU/L)	7.0 ± 2.5	8.0 ± 3.0	0.009 *
Total FSH administered (IU)	2341.9 ± 985.8	2816.6 ± 1130.9	0.00005 *
Retrieved oocytes (*n*)	9.7 ± 6.4	7.2 ± 3.9	0.0003 *
Embryos (*n*)	4.8 ± 3.9	3.4 ± 2.6	0.003 *
Top-quality embryos (%)	16.3 ± 25.2	14.8 ± 23.7	0.5
Usable embryos (%)	66.3 ± 28.1	62.8 ± 33.4	0.4
Implantation rate (%)	23.6	19.1	0.3
Live birth rate/cycle (%)	23.2	18.6	0.3

* significant.
